# A Dioxidovanadium Complex *cis*-[VO2 (obz) py] Attenuates Hyperglycemia in Streptozotocin (STZ)-Induced Diabetic Male Sprague-Dawley Rats via Increased GLUT4 and Glycogen Synthase Expression in the Skeletal Muscle

**DOI:** 10.1155/2022/5372103

**Published:** 2022-01-31

**Authors:** Bonisiwe Mbatha, Andile Khathi, Ntethelelo Sibiya, Irvin Booysen, Phikelelani Ngubane

**Affiliations:** ^1^School of Laboratory Medicine and Medical Sciences, University of KwaZulu Natal, Durban, South Africa; ^2^Pharmacology Division, Faculty of Pharmacy, Rhodes University, Grahamstown, South Africa; ^3^School of Chemistry and Physics, College of Agriculture, Engineering and Sciences, University of KwaZulu-Natal, Pietermaritzburg, South Africa

## Abstract

Vanadium has demonstrated antihyperglycemic effects in diabetes mellitus (DM) but is, however, associated with toxicity. Therefore, new vanadium complexes envisaged to possess heightened therapeutic potency while rendering less toxicity are being explored. Accordingly, the aim of the study was to investigate the effects of a dioxidovanadium (V) complex, cis-[VO2 (obz) py], on selected glucose metabolism markers in streptozotocin (STZ)-induced diabetic rats. STZ-induced diabetic rats were treated orally with cis-[VO2 (obz) py] (10, 20, and 40 mg/kg) twice every 3rd day for 5 weeks. Blood glucose concentrations, body weight, and food and water intake were monitored weekly, for 5 weeks. Rats were then euthanized after which blood, liver, and muscle tissues were collected for biochemical analysis. The administration of dioxidovanadium complex significantly decreased blood glucose concentrations throughout the 5-week period in comparison with the diabetic control (DC). The attenuation of hyperglycemia was accompanied by an increased glycogen concentration in both liver and muscle tissues in the treated groups. Furthermore, a significant increase was observed in the expression of glucose transporter type 4 (GLUT4) in the skeletal muscle tissues and glycogen synthase in the liver tissues. These findings indicate that our vanadium complex cis-[VO2 (obz) py] may exert antihyperglycemic effects through increased glucose uptake, glycogen synthesis, and increased GLUT4 and glycogen synthase expression.

## 1. Introduction

Glucose metabolism is often impaired in diabetes mellitus (DM) due to either the inability of the pancreatic beta cells to produce insulin (type 1) or the inability of insulin to exert its effects (type 2) [1]. According to the World Health Organization (WHO), approximately 422 million people are living with diabetes worldwide [2]. Despite various antidiabetic drugs being used in the management of diabetes, exogenous insulin remains the gold standard treatment for type 1 diabetes [3]. Insulin, an anabolic hormone, plays a major role in maintaining glucose homeostasis through various mechanisms including glucose uptake by the skeletal muscle and the adipose tissues, promoting glycogenesis and inhibiting glycogenolysis and gluconeogenesis [4, 5]. Although insulin administered as a bolus is effective in lowering blood glucose concentrations, it is, however, associated with various complications including hyperinsulinemia, which results in hypoglycemia and increased sodium retention [6, 7]. There is therefore an imperative need to seek alternative treatment that will maintain normoglycemia and attenuate complications associated with the DM.

Transition metals have been extensively investigated for their medicinal benefits and have displayed various effects including antioxidant, anti-inflammatory, and antidiabetic effects [8–10]. Vanadium is a transition metal that has also been reported to possess therapeutic effects including antibacterial, antioxidant, and antidiabetic effects [11, 12]. Although therapeutic effects of vanadium have been investigated for decades and favourable results have been obtained, this transition metal is still not being used in humans for treating diabetes. The major concern about using vanadium for the treatment of a chronic condition such as DM is the toxicity and the tissue accumulation that is associated with prolonged use of this metal. To avert these complications, researchers are focusing on synthesizing organic vanadium complexes, particularly making use of heterocyclic ligands, which have been reported to reduce toxicity, provide stability, and promote bioavailability [13].

Vanadium complexes are reportedly less toxic, more stable, and more effective when compared to the vanadium inorganic salts [14]. Accordingly, we synthesized a dioxidovanadium complex cis-[VO2 (obz) py] using ammonium vanadate (NH_4_VO_3_) and hydroxyphenylbenzimidazole (Hobz) in the presence of pyridine [13]. The use of two organic ligands in the synthesis of this complex may enhance its potency and eliminate the toxicity associated with vanadium. Furthermore, pyridine and benzimidazole are aromatic organic chelators used in the synthesis of many drugs and have also been shown to possess various therapeutic effects including antioxidant activity [15–17]. Hence, this compound may not only alleviate hyperglycemia but may also avert other complications associated with diabetes including oxidative stress. Furthermore, this complex has been shown to improve hepatic function in diabetic rats as evidenced by a decrease in plasma alanine aminotransferase (ALT) and aspartate aminotransferase (AST), which are markers for liver damage [18].

Understanding mechanisms by which this transition metal exerts its antidiabetic effects would play a pivotal role in its development as an alternative antidiabetic agent. Although vanadium complexes have demonstrated antihyperglycemic properties, mechanisms through which these effects are exerted are yet to be fully established. The skeletal muscle is the major site for glucose uptake from the systemic circulation and is often the target of drug development studies. Studies have reported that vanadium has insulin-mimetic effects including the increased expression of GLUT4, which plays a key role in the regulation of glucose homeostasis [11, 19–22]. Accordingly, this study then aimed to evaluate the effects of the dioxidovanadium complex cis-[VO2 (obz) py] on hyperglycemia and establish its mechanism of action in STZ-induced diabetic rats.

## 2. Materials and Methods

### 2.1. Drugs and Chemicals

All drugs and chemicals used were of analytical grade. Dioxidovanadium (V) complex, cis-[VO2 (obz) py], was synthesized as previously described by Dr I Booysen from the Chemistry Department, UKZN, Pietermaritzburg Campus [13]. Briefly, 0.100 g ammonium vanadate (NH_4_VO_3_) was dissolved in 10 cm^3^ of ultrapure water. 0.83 g of 2-hydroxyphenylbenzothiazole (Hobz) dissolved in 10 cm^3^ of methanol was also added, and the solution was heated under reflux for 8 hours. The solution was then allowed to cool to room temperature, and a yellow precipitate was filtered by gravity. This precipitate was then dissolved in a 2-pyridylbenzimidazole (Hpybz) mixture. Yellow needles were isolated from the slow diffusion of ethanol into the solvent mixture. The infrared spectra were recorded in the solid state on a PerkinElmer Spectrum 100 in the 4000–650 cm_1 range. The NMR spectra were obtained using a Bruker Avance 400 MHz Spectrometer. All NMR spectra were recorded in DMSO. The 51 V NMR spectrum was referenced relative to VOCl_3_, while for all 1H NMR spectra, trimethylsilane was used as an internal standard. All chemicals and reagents were of analytical grade and were obtained from Merck Chemicals (Pty) Ltd, Johannesburg, South Africa.

### 2.2. Cytotoxicity Screening

To evaluate the cytotoxic effects of the novel vanadium complex cis-[VO2 (obz) py], we performed a cytotoxicity screening using the 3-(4, 5-dimethylthiazol-2-yl)-2,5-diphenyltetrazolium bromide (MTT) assay. This experiment was conducted using the skeletal muscle (C2C12) cell line. Cell viability was assessed using the MTT assay originally described by Mosmann [23]. Confluent cells were trypsinized and seeded into the 96-well plates (Bibby Sterilin, Staffordshire, England) at a seeding density of 1.8 × 104 cells/well and incubated for 24 h to permit attachment and growth of cells to semi-confluence. Thereafter, the medium (0.2 mL) was replaced with cis-[VO2 (obz) py] (12.5, 25, and 50 *μ*g/mL), which were added to the wells, and cells were incubated at 37°C for 12, 24, and 48 hours, respectively. The cells treated with DMSO (0.1%) served as a control. After each incubation period, the medium was removed and MTT solution (5 mg/mL in phosphate-buffered saline, 200 *μ*L) was added to wells. The cells were incubated for 4 h to allow for the formation of blue formazan crystals. The MTT solution was then replaced with DMSO (200 *μ*L/well), and the absorbance was measured at 570 nm in a UV-visible spectrophotometer (Thermo Scientific Biomate, Cambridge, UK). The percentage cell viability was then calculated as follows:

(1) $$\begin{array}{c} \frac{\left[A570\text{ treated cells}-\text{background}\right]}{\left[A570\text{ control cells}-\text{background}\right]}\times 100. \end{array}$$

### 2.3. Glucose Utilization

To evaluate the effects of the novel vanadium complex cis-[VO2 (obz) py] on glucose uptake in skeletal muscle cell line, we performed the glucose utilization test. The skeletal muscle cell line used in this study was donated by Dr Christo J.F. Muller from the Diabetes Discovery Platform at the South African Medical Research Council (SAMRC), Cape Town, South Africa. The glucose utilization experiments were conducted as previously described by Sibiya [24]. DMEM was used for culturing muscle (C2C12) cell lines and was supplemented with FCS (10%), pen/strep (1%), and L-glutamine (1%). Frozen muscle cell lines were reconstituted in respective media and transferred into 25 cm^3^ flasks, which were incubated at 37°C in the presence of CO_2_ (5%) in a humidified (89%) incubator (Shel Lab, Cornelius, Oregon, USA). The cells were allowed to grow, attach, and become confluent. The attached confluent cells were trypsinized with trypsin (1 mL) after washing three times with PBS. The cells were then subcultured in new flasks and incubated. Thereafter, the cell lines were plated in 24- and 96-well plates for experiments. To prepare doses required for each experiment, vanadium (12.5, 25, and 50 *μ*mol/L) was prepared in DMSO (0.1%) and subsequently diluted in fully supplemented respective cell culture media. At an 80% confluence, the C2C12 cell lines (1.5 × 105) in 24-well plates were incubated at 37°C with DMEM (1 mL) containing 29 mmol/L of glucose in the presence of cis-[VO2 (obz) py] (12.5, 25, and 50 *μ*mol/L). Cells incubated with DMSO (0.1%) and insulin (4 *µ*mol/L) acted as untreated and treated positive controls, respectively. Doses of vanadium complex cis-[VO2 (obz) py] and insulin were determined from a series of preliminary experiments. The cells were passaged at 24 h. Medium glucose concentrations were measured at 0, 12, 24, and 48 h using a glucometer (OneTouch Select Glucometer, LifeScan, Mosta, Malta, UK).

### 2.4. Starch Hydrolysis Enzyme Inhibition

To evaluate the effects of the novel vanadium complex cis-[VO2 (obz) py] on starch hydrolysis enzymes *α*-amylase and *α*-glucosidase, we performed the starch hydrolysis enzyme inhibition assay.

### 2.5. *α*-Amylase

The inhibitory activities of vanadium complex cis-[VO2 (obz) py] on *α*-amylase and *α*-glucosidase were studied using *α*-amylase/*α*-glucosidase starch model, a protocol previously described by Khathi et al. [25], with slight modifications. Appropriate dilutions of 1 mL vanadium complex cis-[VO2 (obz) py] (10, 20, and 30 *µ*g/mL) or acarbose (30 *µ*g/mL) were preincubated with porcine pancreatic *α*-amylase (1 mg/mL) at 25°C for 10 minutes. This was followed by the addition of 1 mL of 1% w/v starch solution in 0.02 M sodium phosphate buffer (pH 6.9 with 0.006 M NaCl to each tube). Thereafter, the reaction mixture was incubated at 25°C for 10 minutes and stopped with 1 mL of dinitrosalicylic acid reagent (2.18 g of 96 mM 3,5-DNSA in 80 mL of 0.5 M NaOH and 30 g of sodium potassium tartrate). The mixture was then incubated in a boiling water bath for 5 minutes and cooled to room temperature. The reaction mixture was diluted by adding 1 mL of distilled water, and absorbance was measured at 540 nm. A blank was prepared without vanadium complex and replaced by equal quantities of buffer at 20°C, and absorbance was measured at 540 nm. The enzyme inhibition rate expressed as percentage of inhibition was calculated using the following formula:

(2) $$\begin{array}{c} \text{inhibition of }\alpha -\text{amylase activity}\left(\text{\%}\right)=\left(\frac{\left(\text{Abs C}-\text{Abs S}\right)}{\text{Abs C}}\right)\times 100, \end{array}$$

where Abs C is the absorbance of the control (100% enzyme activity) and Abs S is the absorbance of the tested sample (vanadium complex or acarbose).

### 2.6. *α*-Glucosidase

200 *µ*L of a crude enzyme solution of rat intestinal *α*-glucosidase, which was adjusted to 0.2 U/mL as an initial concentration in phosphate buffer 67 mM, pH 6.8, was mixed with 200 *µ*L of the sample cis-[VO2 (obz) py] (10, 20, and 30 *µ*g/ml) or acarbose (30 *µ*g/mL) solutions and 1 mL of phosphate buffer. The mixture was preincubated at 37°C for 10 min after which 300 *µ*L of p-NPG solution (10 mM) was added. The reaction mixture was further incubated at 37°C for another 40 min. The reaction was stopped by adding 3 mL of sodium carbonate Na_2_CO_3_ (100 mM). The absorbance of the liberated p-nitrophenol was measured at 405 nm. The enzyme inhibition rate, expressed as a percentage of inhibition, was calculated using the following formula:

(3) $$\begin{array}{c} \text{inhibition of }\alpha -\text{glucosidase activity}\left(\text{\%}\right)=\left(\frac{\left(\text{Abs C}-\text{Abs S}\right)}{\text{Abs C}}\right)\times 100, \end{array}$$

where Abs C is the absorbance of the control (100% enzyme activity) and Abs S is the absorbance of the tested sample (vanadium complex or acarbose).

### 2.7. Animals

Animals used in the study were kept under conditions approved by the University of KwaZulu Natal Animal Research Ethics Committee (AREC). We used male Sprague Dawley rats (250–300 g), bred in the Biomedical Research Unit of the University of KwaZulu Natal. These animals were kept and maintained under standard laboratory conditions (for temperature and humidity) in a 12 h day: 12 h night cycle. The animals were allowed to access water *ad libitum* and were given rat chow daily (Meadow Feeds, Pietermaritzburg, South Africa). Ethical clearance was obtained from the Animal Research Ethics Committee of the University of KwaZulu Natal (AREC/054/017D).

### 2.8. Induction of Diabetes

The induction of type 1 diabetes was carried out using a previously described, well-established protocol [26–28]. Briefly, animals were given a single intraperitoneal injection of STZ (60 mg/kg), freshly prepared in 0.1 M citrate buffer (pH 4.5). The control group was injected with citrate buffer. A week later, fasted animals with blood glucose concentrations greater than 20 mmol/L were considered diabetic. Blood was collected via the tail prick method, and blood glucose concentrations were measured using a glucometer (OneTouch Select Glucometer, LifeScan, Mosta, Malta, UK).

### 2.9. Oral Glucose Tolerance (OGT) Test

To evaluate the short-term effects of the novel vanadium complex on diabetic rats, we performed an oral glucose tolerance (OGT) test. OGT was carried out in all STZ-induced diabetic rats using a previously described protocol [29]. Briefly, separate groups of diabetic rats were fasted overnight (18 hours). This was followed by a glucose loading (0.86 mg/kg) using an 18-gauge gavage needle (Kyron Laboratories (Pty) Ltd, Benrose, South Africa). OGT responses to different concentrations of cis-[VO2 (obz) py] (10.0, 20.0, and 40.0 mg/kg) were monitored. Untreated and insulin-treated rats served as a negative control and a positive control, respectively. Blood was collected via the tail prick method, and blood glucose concentrations were measured using a glucometer (OneTouch Select Glucometer, LifeScan, Mosta, Malta, UK) at 0, 15, 30, 60, 90, and 120 minute intervals.

### 2.10. Short-Term Studies

The experimental animals were divided into 6 groups (*n* = 6 per group) and were housed individually in Makrolon polycarbonate metabolic cages (Tecniplast, Labotec, South Africa). The groups were divided into nondiabetic (ND), diabetic control (DC), diabetic animals treated with subcutaneous insulin (SC Ins), and diabetic animals treated with 3 doses of the vanadium complex (10.0, 20.0, and 40.0 mg/kg, p.o) every 3rd day for 5 weeks using an 18-gauge gavage needle (Kyron Laboratories (Pty) Ltd, Benrose, South Africa). Insulin-treated animals were dosed twice daily for 5 weeks (175 *μ*g/kg, S.C) and served as a positive control. Every 7th day for 5 weeks, blood glucose concentration using the OneTouch Select Glucometer (LifeScan, Mosta, Malta, UK) was monitored using a tail prick method. Body weight, food intake, and water intake were also recorded.

### 2.11. Tissue Sample Harvesting

To harvest blood and tissue samples, all animals were euthanized by exposure to ISOFOR for 3 min via a gas anaesthetic chamber (100 mg kg^−1^). Before they were euthanized, animals had free access to food and water. Blood was collected by cardiac puncture into individual precooled heparinized containers. The liver and skeletal muscle tissues were removed and weighed before freezing in liquid nitrogen. Thereafter, plasma and the tissues were stored in a BioUltra Freezer (Snijders Scientific, Tilburg, Netherlands) at −80°C until use.

### 2.12. Biochemical Analysis

#### 2.12.1. Glycogen Assay

To evaluate the effects of the novel vanadium complex treatment on glycogen synthesis, we carried out a glycogen assay. Glycogen analysis was performed in the cultured skeletal muscle cells harvested at 48 h and in muscle and liver tissues for *in vivo* studies. The glycogen assay was conducted using a well-established laboratory protocol [25, 30, 31]. The harvested skeletal muscle cells, liver, and muscle tissue (50 mg) were heated with KOH (30%, 2 mL) at 100°C for 30 minutes; thereafter, Na_2_SO_4_ (10%, 0.194 mL) was added to stop the reaction and allowed to cool. For glycogen precipitation, the cooled mixture (200 *µ*L) was aspirated and mixed with ethanol (95%, 200 *µ*L). The precipitated glycogen was pelleted, washed, and re-solubilized in H_2_O (1 mL). Thereafter, anthrone (0.5 g dissolved in 250 mL of sulphuric acid, 4 mL) was added and boiled for 10 min. After cooling, the absorbance was read using the SPECTROstar Nano Spectrophotometer (BMG Labtech, Ortenberg, Baden-Württernberg, Germany) at 620 nm. The glycogen concentrations were calculated from the glycogen standard curve. The standard curve ranges from 200 to 1000 mg/L.

### 2.13. Western Blot

Skeletal muscle tissue samples were analysed for the expression of GLUT4 and glycogen synthase by Western blotting, using a protocol previously described by Khathi et al. [25] with slight modifications. Briefly, 0.1 g of muscle tissue was homogenized on ice in RIPA buffer (Bio-Rad, Johannesburg, South Africa) and then centrifuged at 5724 x g for 10 min (4°C). The supernatant was transferred into fresh tubes and centrifuged again at 5724 x g for 10 min (4°C). The supernatant was harvested, and the protein content was quantified using the Bradford assay. Standards were prepared using bovine serum albumin (BSA), and thereafter, serial dilutions were carried out (0.2–1 mg/mL). The standards or samples (100 *μ*L) were mixed with 50 *μ*l of Bradford reagent (Bio-Rad, Johannesburg, South Africa) in a 96-well plate. Thereafter, the plate was incubated at 37°C for 30 minutes, and after cooling, the absorbance was read at 562 nm. The protein concentrations were extrapolated from a protein standard curve (0.2–1 mg/mL). For Western blot analysis, all the protein samples were standardized to 1 mg/mL. The proteins were then denatured by boiling in Laemmli sample buffer (Bio-Rad, Johannesburg, South Africa) for 5 min. The denatured proteins were loaded (20 *μ*L) on prepared resolving (10%) and stacking (4%) polyacrylamide gels. The gels were electrophoresed for 1 h at 150 V in electrode running buffer (Tris base, glycine, SDS, pH 8.3). After electrophoresis, the resolved proteins were electro-transferred to an equilibrated polyvinylidene difluoride (PVDF) membrane for 1 h in transfer buffer (192 mM glycine, 25 mM Tris, 10% methanol). Following the transfer, the membranes were blocked with 5% nonfat dry milk in Tris-buffered saline with 0.1% Tween-20 (TTBS) (20 mM Tris, 150 mM NaCl, KCl, 0.05% Tween-20). The membranes were then immuno-probed with antibodies GLUT4 (anti-rabbit, polyclonal, catalogue number: 07–1404; Sigma-Aldrich, Johannesburg, South Africa) and glycogen synthase (anti-mouse, monoclonal, catalogue number: VMA00048; Bio-Rad, Johannesburg, South Africa) (1:1000 in 1% TTBS) for 1 h at room temperature. Beta-actin antibody (Bio-Rad, Johannesburg, South Africa) was used as a loading control. The PVDF membrane was then subjected to 5 washes (10 min with gentle agitation) with TTBS. The membranes were then incubated in HRP-conjugated secondary antibody (rabbit anti-mouse 1:1000 in TTBS) for 1 h at room temperature. After washing, antigen-antibody complexes were detected by chemiluminescence using the Immun-Star HRP Substrate Kit (Bio-Rad, Johannesburg, South Africa). Chemiluminescent signals were detected with the Chemi-Doc XRS gel documentation system and analysed using the Quantity One software (Bio-Rad, Johannesburg, South Africa). Band intensity analysis was conducted on the resultant bands.

### 2.14. Statistical Analysis

All data are expressed as mean ± standard error of means (SEM). Statistical analysis was performed using GraphPad Prism Software (version 5). Data for bodyweight, food intake, and water intake were analysed using the two-way repeated-measures analysis of variance (ANOVA), which were followed by the Bonferroni post hoc test. All other data analysis was performed using the one-way ANOVA, which was followed by the Tukey–Kramer post hoc test for analysis of differences between the control and experimental groups. The values of *p* < 0.05 were considered statistically significant between the compared groups.

## 3. Results

### 3.1. In Vitro Studies

#### 3.1.1. Cell Viability

**Figure 1:**
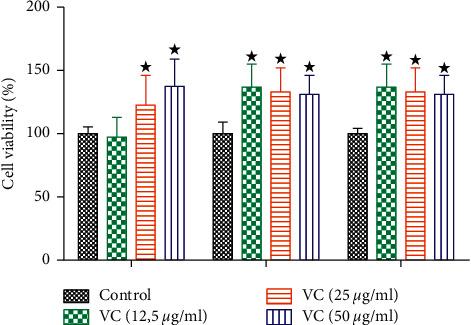
The effects of vanadium complex (VC) on cell viability in skeletal muscle cells at 12, 24, and 48 h. The values are presented as means, and vertical bars indicate SEM (*n* = 6 in each group). ^★^*p* < 0.05 by comparison with the control group at each corresponding time.

To evaluate the cytotoxic effects of the novel vanadium compound, we treated the C1C12 skeletal muscle cell line with cis-[VO2 (obz) py] (12.5, 25, and 50 *μ*g/mL) (Figure 1). The administration of all 3 doses of our vanadium complex showed no cytotoxic effect in the cells in comparison with the control at time intervals of 12, 24, and 48 h, respectively.

#### 3.1.2. Medium Glucose in Skeletal Muscle Cells

**Figure 2:**
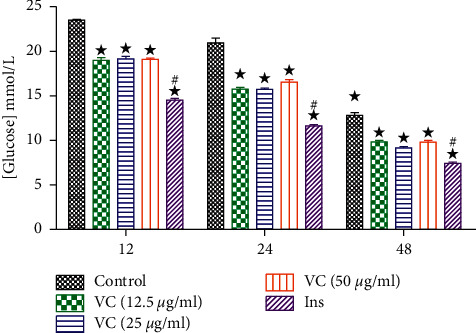
The effects of vanadium complex (VC) and insulin (Ins) on medium glucose in skeletal muscle cells at 12, 24, and 48 h. The values are presented as means, and vertical bars indicate SEM (*n* = 6 in each group). ^★^*p* < 0.05 by comparison with control groups at each corresponding time. ^#^*p* < 0.05 by comparison with all vanadium doses at each corresponding time.

To evaluate the effect of vanadium complex on glucose uptake by the cells, we treated the skeletal muscle cells with cis-[VO2 (obz) py] (12.5, 25, and 50 *μ*g/mL).  Figure 2 shows the effects of this complex on glucose utilization at 12, 24, and 48 h in skeletal muscle cells. The control group showed a steady decline in glucose concentrations over 48 h experimental period. The vanadium complex was able to significantly decrease medium glucose concentrations in comparison with the control (*p* < 0.05). However, there was no dose-dependent effect among the 3 doses. Similarly, insulin administration also significantly decreased medium glucose in comparison with the control at 12, 24, and 48 h.

#### 3.1.3. Inhibition of *α*-Amylase and *α*-Glucosidase

We evaluated the inhibitory effects of the novel vanadium compound on starch hydrolysis enzymes *α*-amylase and *α*-glucosidase.  Table 1 shows the percentage inhibition of *α*-amylase and *α*-glucosidase by acarbose (30 *µ*g/mL) and vanadium complex cis-[VO2 (obz) py] (10, 20, and 30 *µ*g/mL). The vanadium complex inhibited *α*-amylase and *α*-glucosidase with IC_50_ values of 34.41 ± 1.27 and 17.49 ± 0.83, respectively. Our standard drug acarbose inhibited *α*-amylase and *α*-glucosidase with IC_50_ values of 30.64 ± 1.08 and 12.7 ± 0.74, respectively.

### 3.2. Animal Studies

#### 3.2.1. OGT Responses

**Figure 3:**
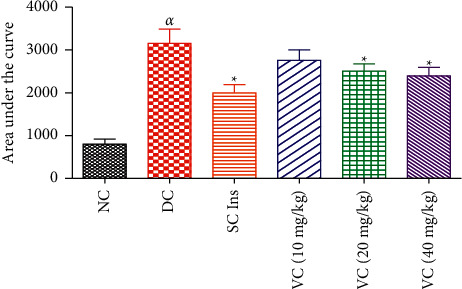
AUC calculated from the OGTT of nondiabetic control (NC), diabetic control (DC), and diabetic rats treated with the vanadium complex (VC) (10, 20, and 40 mg/kg) and insulin (SC Ins). *αp* < 0.05 by comparison with the NC. ^∗^*p* < 0.05 by comparison with the DC.

Figure 3 shows the OGT responses, which were analysed by calculating the area under the curve (AUC) of the nondiabetic animals (NC), diabetic control (DC), and diabetic animals treated with the vanadium complex (VC) (10, 20, and 40 mg/kg) and insulin (175 *μ*g/kg, SC). High glucose concentrations were observed in the STZ-induced diabetic control compared with the NC. All 3 doses of VC significantly decreased blood glucose concentration in comparison with the DC (*p* < 0.05). Insulin also significantly decreased blood glucose concentrations in comparison with STZ-induced untreated rats (*p* < 0.05).

#### 3.2.2. Weekly Blood Glucose Concentration

**Figure 4:**
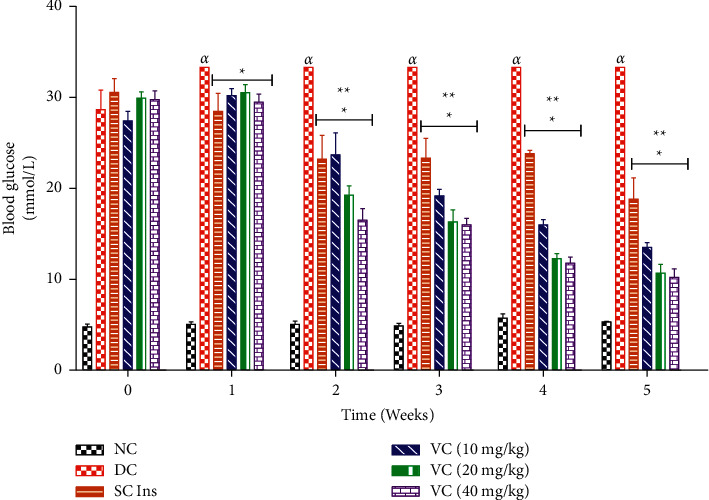
Blood glucose concentrations of the nondiabetic control (NC), diabetic control (DC), and diabetic rats treated with the vanadium complex (VC) (10, 20, and 40 mg/kg) and insulin (SC Ins). *αp* < 0.05 by comparison with the NC. ^*∗*^*p* < 0.05 by comparison with the DC. ^*∗∗*^*p* < 0.05 by comparison with baseline.

Blood glucose concentrations were monitored in NC, DC, and diabetic animals treated with VC and SC Ins (Figure 4). The DC group showed elevated blood glucose concentrations throughout the 5-week period in comparison with the NC (Figure 4, DC vs ND, *p* < 0.05). Treatment with all 3 doses of VC was able to significantly decrease blood glucose concentrations in comparison with the DC (Figure 4, DC vs VC, *p* < 0.05) with a significant decrease observed from week 2 onwards in comparison with baseline. Subcutaneous insulin treatment also demonstrated a significant decrease in blood glucose concentrations when compared to the DC (Figure 4, DC vs SC, *p* < 0.05).

#### 3.2.3. Body Weight, Food Intake, and Water Intake

**Figure 5:**
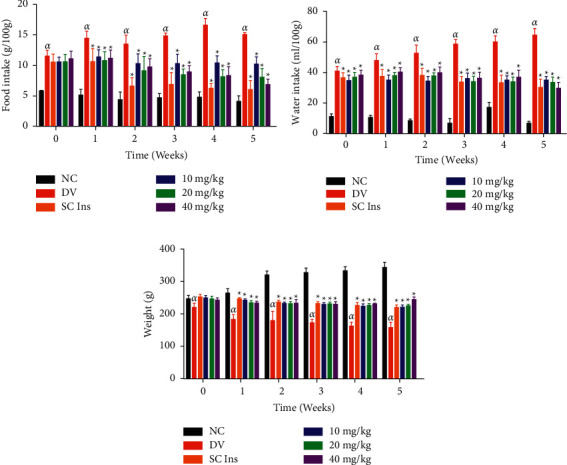
Water intake (a), food intake (b), and bodyweight change (c) in nondiabetic control (ND), diabetic control (DC), and diabetic animals treated with subcutaneous insulin (SC Ins 175 *μ*g/kg) and vanadium complex (VC) (10, 20, and 40 mg/kg). Data are expressed as mean ± SEM, *n* = 6 in each group. *αp* < 0.05 by comparison with the NC. ^★^*p* < 0.05 by comparison with the DC.

Figure 5 compares food intake, water intake, and body weight changes in the nondiabetic, untreated STZ-induced diabetic, and insulin- and vanadium-treated STZ-induced diabetic rats over a period of five weeks. The untreated STZ diabetic rats exhibited a progressive weight loss throughout the 5-week treatment period accompanied by significantly high food and water intake in comparison with the nondiabetic control group (*p* < 0.05). The vanadium-treated animals, however, showed a significant decrease in food intake while increasing body weight and water intake in comparison with the untreated diabetic animals. A progressive decrease in body weight change was observed in animals treated with insulin and those treated with vanadium over a period of 5 weeks. A sharp decline in food intake of animals treated with insulin was observed at week 2, and it is remained at normal levels throughout the treatment period.

### 3.3. Biochemical Studies

#### 3.3.1. Glycogen Concentrations

Table 2 compares glycogen concentrations of nondiabetic control (ND), diabetic control (DC), and STZ-induced diabetic rats treated with subcutaneous insulin (SC Ins 175 *μ*g/kg) and vanadium complex cis-[VO2 (obz) py]. There was a significant decrease in glycogen concentrations in both muscle and liver tissues of the DC when compared to the NC group at the end of our treatment period. Treatment with the vanadium complex resulted in a significant increase in muscle and liver glycogen content when compared with the DC group.

#### 3.3.2. Western Blot

**Figure 6:**
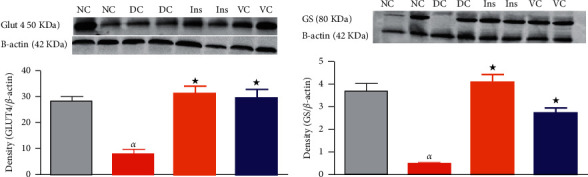
GLUT4 expression in skeletal muscle (a), glycogen synthase expression in the liver (b) of nondiabetic control (NC), diabetic control (DC), and diabetic animals treated with insulin (Ins) and vanadium complex (VC) (40 mg/kg). Data are expressed as mean ± SEM, *n* = 6 in each group. ^★^*p* < 0.05 by comparison with the DC. *αp* < 0.05 by comparison with the NC.

The effects of the novel vanadium complex cis-[VO2 (obz) py] on the expression of GLUT4 and GS were evaluated using the Western blot technique (Figure 6).  Figure 6(a) shows the expression of GLUT4 in skeletal muscle tissues of the nondiabetic control (NC), diabetic control (DC), and STZ-induced diabetic rats treated with subcutaneous insulin (SC ins) and vanadium complex cis-[VO2 (obz) py]. There was a significant decrease in GLUT4 expression of the nontreated diabetic rats in comparison with the nondiabetic control. The animals treated with the vanadium complex (40 mg/kg) showed an increase in GLUT4 expression, which was evidenced by a strong band intensity in comparison with the diabetic control. The animals treated with insulin (SC Ins175 *μ*g/kg) also showed an increase in GLUT4 expression in comparison with the DC.

Figure 6(b) shows the expression of glycogen synthase in liver NC, DC, and STZ-induced diabetic rats treated with SC Ins and VC. There was a significant decrease in glycogen synthase expression of the untreated diabetic rats in comparison with NC. However, the animals treated with VC (40 mg/kg) displayed an increase in glycogen synthase expression in the liver tissues in comparison with DC.

## 4. Discussion

This study evaluated the effects of a dioxidovanadium complex cis-[VO2 (obz) py] on glucose metabolism and the possible mechanisms by which this metal complex may exert these effects in STZ-induced diabetic male Sprague Dawley rats. Due to the reported toxicity of vanadium, the first study undertaken was the screening of this complex for cytotoxicity in the skeletal muscle cell line. Studies have shown various toxic effects of vanadium salts including the necrosis in the small intestine and renal and hepatic lesions [32–34]. The side effects of orally administered vanadium include gastrointestinal problems such as vomiting, diarrhoea, dehydration, and also weight loss [35]. Research has demonstrated, however, that the synthesis of organic vanadium complexes reduces toxicity and increases bioavailability, making the vanadium complexes more effective and safer compared with the vanadium salts [14, 36]. This dioxidovanadium complex was synthesized using NH_4_VO_3_ and hydroxyphenylbenzimidazole (Hobz) in the presence of pyridine. Benzimidazole is an organic ligand that has been wildly used in the synthesis of various drugs and metal complexes. This bicyclic ligand possesses biological benefits including antioxidant and antibacterial activities, and its use in the synthesis of compounds has been shown to improve activity and bioavailability [37, 38].

Pyridine is also a widely used heterocyclic ligand in the synthesis of various drugs. Its various ring positions reportedly make it a preferred building block for drug synthesis and result in improved stability and bioavailability of the resultant compounds [17]. This vanadium complex exhibited no cytotoxic effects in the skeletal muscle cell line indicating that the use of heterocyclic ligands may have curbed vanadium toxicity. However, the narrow ranges between the doses used in this experiment may have limited the observations. We have also shown that this novel compound has hepatoprotective effects through amelioration of oxidative stress in the liver and decreasing liver enzyme AST and ALT concentrations in STZ-induced diabetic rats [18]. Furthermore, the animals treated with cis-[VO2 (obz) py] did not present with any side effects and showed improved body weight compared with the diabetic animals. Therefore, these observations suggest that we may have been successful in mitigating or curbing the toxicity hazards associated with vanadium through making use of the organic ligands. The absence of side effects combined with the alleviation of hyperglycemia suggests that the strategy employed in the synthesis of our vanadium complex attenuated the vanadium toxicity without altering its antidiabetic effects.

Studies report that vanadium's half-life in the systemic circulation ranges from 2 to 12 days depending on the administered dose and the route of administration [39, 40]. Vanadium is also reported to accumulate in tissues of organs such as the kidneys, which may alter their function. To avert complications associated with the accumulation of vanadium, our experimental animals were treated with the vanadium complex every 3rd day for 5 weeks in contrast with various studies, whereby animals are treated with vanadium daily. Hence, this treatment schedule may have also circumvented undesirable effects.

The antidiabetic effects of vanadium complexes have been extensively reported in both *in vitro* and *in vivo* [41–43]. Studies carried out by Willsky et al. [44] showed that a series of vanadium dipicolinate complexes ameliorated hyperglycemia in STZ-induced diabetic rats. Similarly, we have shown that cis-[VO2 (obz) py] is able to lower blood glucose concentration in both acute and chronic studies in comparison with the untreated control groups. This further validates documented antihyperglycemic effects of vanadium complexes. Vanadium has been reported to protect and regenerate pancreatic beta cells in STZ-induced diabetic rats [45, 46]. Missaoiu et al. [45] showed that diabetic rats treated with vanadium presented with near-normal insulin concentrations at the end of the treatment period. Hence, future studies should measure plasma insulin concentrations to elucidate whether the hyperglycemic effects observed in this study are conferred by cis-[VO2 (obz) py] alone and not insulin.

To understand the mechanisms by which this complex decreases blood glucose concentrations, we evaluated its effects on glucose uptake in the skeletal muscle cell line. The ability of our vanadium complex to decrease medium glucose concentrations in skeletal muscle cell line indicates that skeletal muscle glucose uptake may be one of the mechanisms by which our compound exerts antihyperglycemic effects. However, the lack of the dose-dependent response among the 3 doses of the vanadium complex observed in this study could be due to the narrow range between the doses. This could also be as a result of the drug transporter having reached saturation as the starting dose of 12.5 *µ*L may have been too high. Glucose uptake in the skeletal muscle is facilitated through the translocation of glucose transporter 4 (GLUT4) from the vesicles to the plasma membrane, a process initiated by the binding of insulin to the alpha subunit of the insulin receptor. Vanadium has been reported to increase GLUT4 expression, thereby facilitating glucose disposal in skeletal muscle. Vanadium complexes such as bis(maltolato)oxovanadium (BMOV) and bisperoxovanadium were shown to increase GLUT4 expression in cardiac and skeletal muscles, respectively [21, 47]. Similarly, our complex increased GLUT4 expression in skeletal muscle tissues compared with the DC. These findings, therefore, indicate that increased GLUT4 expression may be one of the mechanisms employed by this vanadium compound in attenuating hyperglycemia. However, GLUT4 expression was measured in the whole skeletal muscle tissue. Hence, these results only indicate the increased expression of this transporter and not necessarily its translocation from the vesicles to the apical membrane. Further studies identifying the translocated GLUT4 should be carried out to further elucidate the role of vanadium in this pathway. However, a decrease in media and blood glucose *in vitro* and *in vivo,* respectively, indicates that this vanadium complex may increase glucose uptake through the translocation of GLUT4.

Increased glycogen synthesis via the activation of glycogen synthase, a key enzyme in glycogen synthesis, is one of the mechanisms through which insulin exerts antihyperglycemic effects [48]. Accordingly, we evaluated the effects of this vanadium complex on glycogen synthesis in the liver and skeletal muscle tissues. An increase in glycogen concentrations in both skeletal muscle and liver tissues of animals treated with cis-[VO2 (obz) py] was observed. This perhaps explains the increase in glucose utilization observed in the *in vitro* studies, where skeletal muscle cells were treated with vanadium complex, and also in congruence with blood glucose concentration observation. Semiz et al. [49] reported that vanadium did not stimulate glycogen synthesis or glycogen synthase concentrations in skeletal muscle. However, a study carried out by Ramachandran et al. [50] demonstrated that macrocyclic binuclear oxovanadium complexes increased the activity of glycogen synthase and glycogen content in diabetic rats. Similarly, cis-[VO2 (obz) py] increased glycogen synthase expression in the liver tissues, which is in line with the growing body of literature that suggests that glycogen synthesis may be another mechanism by which vanadium exerts antihyperglycemic effects [51]. Glycogen content in this study was measured on fed rats, and hence, studies where glycogen quantity is measured on fasted rats may help elucidate the exact role of vanadium on glycogen synthesis. Vanadium has been reported to interact with the actin cytoskeleton resulting in decreased actin concentration [52, 53]. This might have an impact on our GLUT4 and GS expression results since *β*-actin was used as a housekeeping antibody. Studies investigating the effects of vanadium on actin cytoskeleton are seldom. Hence, it is important to note that in the animal studies where actin was affected, the route of vanadium administration was inhalation [53]. The liver has been reported to be one of the organs affected by inhaled vanadium toxicity, but we could not find studies where the skeletal muscle was affected [54]. Therefore, the use of a different loading control such as beta 2-microglobulin may clarify whether actin had any influence on our observations.

Postprandial hyperglycemia reportedly plays a significant role in the pathogenesis of various diabetic complications including atherosclerosis [55]. Pancreatic *α*-amylase and mucosal *α*-glucosidase are enzymes responsible for digesting starch into glucose, which results in a spike in blood glucose concentrations following the consumption of a meal. Inhibition of these starch hydrolysis enzymes has been shown to attenuate postprandial hyperglycemia [56, 57]. Hence, various studies are exploring this mechanism as one of the potential strategies to manage DM. As a result, drugs such as acarbose, voglibose, and metformin abolish hyperglycemia through the inhibition of these enzymes, thus delaying glucose absorption in the small intestine. The inhibition of alpha-amylase and alpha-glucosidase by our vanadium complex in vitro may further explain vanadium's antihyperglycemic properties, and this study is the first to report on such. These observations may also in part explain the reduction in food intake in vanadium complex treated animals; however, we do not exclude other possibilities.

## 5. Conclusions

In conclusion, the novel dioxidovanadium complex cis-[VO2 (obz) py] proved to be noncytotoxic and was able to lower blood glucose concentrations in diabetic animals. Furthermore, the antihyperglycemic properties observed may be mediated through enhanced glucose uptake via increased GLUT4 and glycogen synthase expression in the skeletal muscle and the liver. These observations encourage further research and developments to enhance the overall therapeutic potency of this compound against diabetes mellitus.

## Figures and Tables

**Table 1:** Percentage inhibition of *α*-amylase and *α*-glucosidase by vanadium complex cis-[VO2 (obz) py] (VC) (10, 20, and 30 *µ*g/ml) and acarbose (30 *µ*g/ml).

Treatment	*α*-Amylase % inhibition	*α*-Glucosidase % inhibition
Control	0.00 ± 0.0	0.00 ± 0.0
VC 10 *µ*g/ml	16.3 ± 6.1^#^	27.1 ± 1.7^#^
VC 20 *µ*g/ml	33.3 ± 2.1^#^	47.5 ± 0.9^#^
VC 30 *µ*g/ml	37.1 ± 0.7^#^	53.5 ± 0.9^#^
Acarbose 30 *µ*g/ml	53.5 ± 0.9^#^	55.7 ± 0.8^#^

^#^ *p* < 0.05 by comparison with the control.

**Table 2:** Liver and muscle glycogen measurements of nondiabetic control (ND), diabetic control (DC), and diabetic animals treated with insulin (SC Ins 175 *μ*g/kg) and vanadium complex (VC).

Experimental groups	Glycogen muscle (nmol/g)	Glycogen liver (nmol/g)
NC	0.13 ± 0.01	0.19 ± 0.03
DC	0.07 ± 0.00^**#**^	0.10 ± 0.01^**#**^
SC Ins (175 *μ*g/kg)	0.12 ± 0.01^★^	0.17 ± 0.03^★^
VC (40 mg/kg)	0.11 ± 0.01^★^	0.14 ± 0.00^★^

^★^ *p* < 0.05 by comparison with the DC. ^#^*p* < 0.05 by comparison with the NC. Data are expressed as mean ± SEM, *n* = 6 in each group.

## Data Availability

Additional data are available upon request.
